# White Matter Correlates of Auditory Comprehension Outcomes in Chronic Post-Stroke Aphasia

**DOI:** 10.3389/fneur.2017.00054

**Published:** 2017-02-22

**Authors:** Shihui Xing, Elizabeth H. Lacey, Laura M. Skipper-Kallal, Jinsheng Zeng, Peter E. Turkeltaub

**Affiliations:** ^1^Department of Neurology, First Affiliated Hospital of Sun Yat-Sen University, Guangzhou, China; ^2^Department of Neurology, Georgetown University Medical Center, Washington, DC, USA; ^3^Research Division, MedStar National Rehabilitation Hospital, Washington, DC, USA

**Keywords:** aphasia, stroke, white matter, diffusion tensor imaging, outcome

## Abstract

Neuroimaging studies have shown that speech comprehension involves a number of widely distributed regions within the frontal and temporal lobes. We aimed to examine the differential contributions of white matter connectivity to auditory word and sentence comprehension in chronic post-stroke aphasia. Structural and diffusion MRI data were acquired on 40 patients with chronic post-stroke aphasia. A battery of auditory word and sentence comprehension tests were administered to all the patients. Tract-based spatial statistics were used to identify areas in which white matter integrity related to specific comprehension deficits. Relevant tracts were reconstructed using probabilistic tractography in healthy older participants, and the mean values of fractional anisotropy (FA), mean diffusivity (MD), axial diffusivity (AD), and radial diffusivity (RD) of the entire tracts were examined in relation to comprehension scores. Anterior temporal white matter integrity loss and involvement of the uncinate fasciculus related to word-level comprehension deficits (*R*_FA_ = 0.408, *P* = 0.012; *R*_MD_ = −0.429, *P* = 0.008; *R*_AD_ = −0.424, *P* = 0.009; *R*_RD_ = −0.439, *P* = 0.007). Posterior temporal white matter integrity loss and involvement of the inferior longitudinal fasciculus related to sentence-level comprehension deficits (*R*_FA_ = 0.382, *P* = 0.02; *R*_MD_ = −0.461, *P* = 0.004; *R*_AD_ = −0.457, *P* = 0.004; *R*_RD_ = −0.453, *P* = 0.005). Loss of white matter integrity in the inferior fronto-occipital fasciculus related to both word- and sentence-level comprehension (word-level scores: *R*_FA_ = 0.41, *P* = 0.012; *R*_MD_ = −0.447, *P* = 0.006; *R*_AD_ = −0.489, *P* = 0.002; *R*_RD_ = −0.432, *P* = 0.008; sentence-level scores: *R*_FA_ = 0.409, *P* = 0.012; *R*_MD_ = −0.413, *P* = 0.011; *R*_AD_ = −0.408, *P* = 0.012; *R*_RD_ = −0.413, *P* = 0.011). Lesion overlap, but not white matter integrity, in the arcuate fasciculus related to sentence-level comprehension deficits. These findings suggest that word-level comprehension outcomes in chronic post-stroke aphasia rely primarily on anterior temporal lobe pathways, whereas sentence-level comprehension relies on more widespread pathways including the posterior temporal lobe.

## Introduction

The era of modern neuroimaging has clarified that language extends well beyond the classical network composed of Wernicke’s area, the arcuate fasciculus (AF), and Broca’s area (1, 2). The putative location of Wernicke’s area has shifted over time, and recent studies suggest that auditory comprehension of words relies on cortex considerably anterior to the traditional location of Wernicke’s area in the posterior temporal lobe (3). A prominent study recently examined patterns of cortical atrophy in primary progressive aphasia, finding an anatomical dissociation, in which word-level auditory comprehension related to cortical thinning in the anterior temporal lobe, whereas sentence-level auditory comprehension related to thinning in a more widespread network of posterior temporal and frontal sites (4). The differences in localization of auditory comprehension in primary progressive aphasia from the classical localization based on stroke lesions were attributed to the white matter damage associated with stroke.

Indeed, language processing depends not only on cortical regions but also on the white matter fiber bundles that connect them (5). Recent diffusion tensor imaging (DTI) studies have suggested that the dual streams connecting frontal and temporal language areas differentially support particular aspects of language comprehension processing in the context of healthy participants (6, 7) and pathological conditions such as primary progressive aphasia (8). Only a few studies have used DTI to analyze white matter damage in post-stroke aphasia (9–12), and these have not come to a consensus regarding the contributions of specific tracts to particular auditory comprehension impairments. This likely relates to the small number of participants in these studies, and the investigation of either word- or sentence-level comprehension, but not both. Further, some these prior studies only either examined direct lesion load in tracts, or assessed relationships between comprehension and certain DTI metrics of white matter in the tract (9, 10). As such, the contributions of specific white matter tracts to different aspects of auditory language comprehension remain incompletely understood.

In the present study, we adopted tract-based spatial statistics (TBSS) to identify critical regions of white matter lesions related to severity of word- and sentence-level comprehension deficits in chronic post-stroke aphasia. We then used probabilistic DTI tractography in a group of age-matched healthy older participants to reconstruct specific white matter tracts implicated by TBSS and examined the relationship between the integrity of these tracts in patients and both word- and sentence-level comprehension. We hypothesized that different ventral or dorsal white matter tracts sustain different aspects of comprehension in chronic post-stroke aphasia, in particular that word-level comprehension deficits relate to anterior temporal lobe white matter damage, whereas sentence-level comprehension deficits relate to more posterior temporal lobe white matter damage.

## Materials and Methods

### Participants

Forty (14 females) chronic left hemisphere stroke survivors were recruited in the study with inclusion criteria as follows: native English speaker; at least 6 months post-stroke; able to follow testing instructions; and no history of other significant neurological illnesses. Demographics and language scores for the group are listed in Table 1. All patients had aphasia at the time of stroke based on medical records and received speech–language therapy.

**Table 1:** **Demographic data and language measures in patients**.

Variables	Patients
Age at screening (years)	59.6 ± 10.1
Gender (F/M)	14/26
Education level (years)	16.3 ± 2.9
Handedness (R/L/ambidextrous)	33/6/1
Lesion volume (ml)	101.1 ± 74.1
Time from stroke (months)	45.3 ± 38.6
Language tests	
Word-level composite	0.91 ± 0.12 (0.45–1.00)
Word recognition (60)^a^	53.18 ± 9.31 (27–60)
Word-to-picture matching (48)	44.53 ± 5.32 (22–48)
Sentence-level composite	0.77 ± 0.16 (0.45–0.99)
AVCom Yes/No (60)^a^	55.43 ± 5.04 (39–60)
AVCom sequential commands (80)^a^	57.28 ± 18.50 (10–80)
Complex ideational materials (12)^b^	7.90 ± 3.09 (2–12)
Semantic total scores (60)^b^	54.68 ± 5.47 (34–60)
Embedded sentences (10)^b^	6.38 ± 2.72 (1–10)
Pyramids and Palm Trees test	44.28 ± 4.76 (27–49)

*F, female; M, male; R, right-handed; L, left-handed; AVCom, auditory-verbal comprehension*.

*^a^Western Aphasia Battery-Revised*.

*^b^Boston Diagnostic Aphasia Evaluation*.

*For each language test, the maximum score is shown in brackets*.

Probabilistic tract reconstruction was performed in 27 healthy older participants (16 female) without neurological and psychiatric disorder. The mean age was 59.8 (±14.3) years; mean education was 16.3 (±2.6) years. Among them, 24 were right-handed, 2 left-handed, and 1 ambidextrous.

The study was approved by the Georgetown University Institutional Review Board, and written informed consent was obtained from all study participants.

### Language Comprehension Assessment and Composite Scores

Word-level auditory comprehension was tested with the Auditory-Verbal Comprehension Word Recognition subtest of the Western Aphasia Battery-Revised (WAB-R) (13) and an in-house word-to-picture matching task, in which the tester speaks a word aloud and the participant must select a picture corresponding to the word from among five semantic foils presented on a computer screen. There are 48 trials, and all words are concrete nouns. There is no time limit for response. The test was previously normed on 22 healthy elderly participants matched for age and education (14). For sentence comprehension assessment, the tests included the Auditory-Verbal Comprehension Yes/No and Sequential Commands subtests of the WAB-R, and the Boston Diagnostic Aphasia Examination subtests: complex ideational materials, semantic probe, and embedded sentences tasks. Using these scores, we calculated word-level comprehension (WLC) and sentence-level comprehension (SLC) composite scores to provide robust measurements of these two aspects of comprehension. Raw scores were normalized by dividing by the maximum score of each task, and these normalized scores for each component of comprehension were averaged. Because sentence-level comprehension encompasses word-level comprehension, deficits in either could cause decreased SLC scores. Thus, the WLC scores were regressed out of the SLC scores to obtain a final residual SLC score that controls for word comprehension deficits. Non-verbal semantic performance was additionally assessed with the Pyramids and Palm Trees (PPT) test (15).

### Image Acquisition

Subjects were scanned on a 3.0-T Siemens Trio scanner at the Georgetown University Medical Center. The high-resolution T1-weighted images were acquired with the following parameters: TR = 1,900 ms; TE = 2.56 ms; flip angle = 9°; 160 contiguous 1 mm sagittal slices; field of view = 250 mm × 250 mm; matrix size = 246 × 256, voxel size = 1 mm × 1 mm × 1 mm; slice thickness = 1 mm. Diffusion data were acquired using a single-shot echo-planar imaging sequence with the following parameters: TR = 7,500 ms; TE = 87 ms; flip angle = 90°; field of view = 240 mm × 240 mm; matrix size = 96 × 96, voxel size = 2.5 mm × 2.5 mm × 2.5 mm; slice thickness = 2.5 mm; sagittal slice number = 64 slices. Sixty diffusion volumes weighted with a *b*_max_ value of 1,100 s/mm^2^ and 10 volumes with no diffusion gradient were acquired. An additional 10 volumes with a low diffusion gradient (*b*_min_ = 300 s/mm^2^) were collected to reconstruct a diffusion tensor from the combination of volumes with *b*_min_ and *b*_max_ diffusion weighting, which can reduce the vascular contribution to the diffusion parameters as well as partial volume effects as a result of the signal intensity of CSF (16–18).

### Imaging Data Preprocessing

#### Structural MRI Lesion Delineation

**Figure 1:**
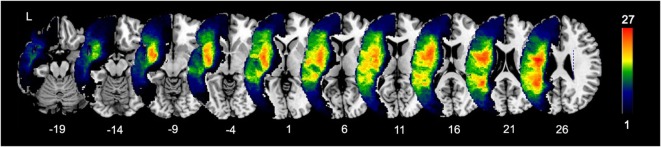
**Lesion overlap of 40 patients**. Color bar indicates the number of patients with lesions in each voxel (maximum 27 out of 40).

Lesion masks of patients were created by manually tracing stroke damage on the native 3D T1 images by using MRIcron (19). Lesion masks were checked by two board-certified neurologists (Shihui Xing and Peter E. Turkeltaub). Structural T1 images were registered to a Montreal Neurological Institute (MNI) brain template in standard space using the unified segmentation tools implemented in Statistical Parametric Mapping software (SPM8)^1^ running under Matlab R2014a (20). Deformations were then applied to warp the lesion masks into the MNI space (21). An overlap of patients’ lesions is shown in Figure 1.

#### Diffusion MRI Lesion Analysis

Preprocessing of the diffusion images was performed using the FMRIB Software Library (FSL).^2^ For each subject, the DWI datasets were preprocessed using FSL diffusion toolbox. Eddy current distortions and motion artifacts were corrected by registering each diffusion volume to the non-diffusion volume with an affine transform. Whole-brain maps of voxelwise DTI metrics were extracted including fractional anisotropy (FA), mean diffusivity (MD), axial diffusivity (AD), and radial diffusivity (RD).

### Tract-Based Spatial Statistical Analysis

Whole-brain voxelwise analyses of the respective DTI metrics data were carried out with TBSS analyses (22). Briefly, FA maps were aligned to an averaged FA template in MNI standard space using a non-linear registration with FNIRT in FSL and individual lesion drawings were employed as masks to avoid deformation of the lesion area. An average FA map was created and a skeleton map representing the center of the white matter common to all patients was computed with a threshold at 0.2. All registered FA maps were finally projected into the skeleton. The same procedure was performed on MD, AD, and RD maps. Controlling for age and total lesion volume, separate regression analyses were conducted to identify voxels in which the white matter signal related to WLC and SLC with lesion masks to exclude lesioned voxels in each patient. All statistics were performed with 5,000 random permutations and a threshold-free cluster enhancement correction (23). Results were family wise error corrected and thresholded at *P* < 0.05.

### Tract-Specific Analysis

To examine relationships of more comprehensive measures of entire tracts to behavioral performance, we conducted a tract-specific analysis. To precisely localize relevant tracts, we reconstructed the left inferior fronto-occipital fasciculus (IFOF), inferior longitudinal fasciculus (ILF), and uncinate fasciculus (UF) tracts implicated by the TBSS analysis (see Results) in 27 age-matched healthy subjects. For completeness, we also reconstructed the major dorsal language stream tract (AF) as well. Paired regions in the left hemisphere was defined as “seed” and “target” to reconstruct connections between these areas as previously described (24, 25). The regions were reversely transferred to the subject’s native DWI space using non-linear registrations for FA images. Fiber tracking was performed using a probabilistic tractography algorithm based on Bayesian estimation of diffusion parameters (26). Fiber tracking was initiated from all voxels within the seed masks in the diffusion space to generate 5,000 streamline samples, with a step length of 0.5 mm and a curvature threshold of 0.2. The resultant tract density maps were normalized by dividing by the total number of streamline samples and thresholded at 0.005 to exclude spurious connections. Tracts were then non-linearly registered to MNI space using the registration parameters for FA images as above. The final groupwise probability map for each tract was generated with a threshold at least 50% (14/27 = 0.52) of healthy subjects (27, 28), and the respective binary map was generated as template to extract the mean values of DTI metrics for individual patient.

### Statistical Analysis

To assess brain–behavior associations in the tracts identified above, we performed partial correlations between the mean values of FA, MD, AD, and RD (as indexes of white matter integrity) and WLC and SLC scores (as indexes of different level in comprehension processing) controlling for age and total lesion volume. Direct lesion volume within a tract may dramatically alter the mean values of the white matter integrity measures and could also covary with direct damage in neighboring tracts or gray matter yielding spurious tract–behavior relationships. Therefore, we further excluded the primary lesion load on each tract from analyses by including the number of lesioned voxels in the tracts as nuisance covariates in the analyses. Statistical analyses were performed using SPSS (version 22). As previously described (29), we considered tracts that showed significant correlations with behavior for the each DTI index at a threshold of *P* < (0.05/4) = 0.0125 (Bonferroni correction).

## Results

### Localization of White Matter Correlates of Comprehension Deficits

**Figure 2:**
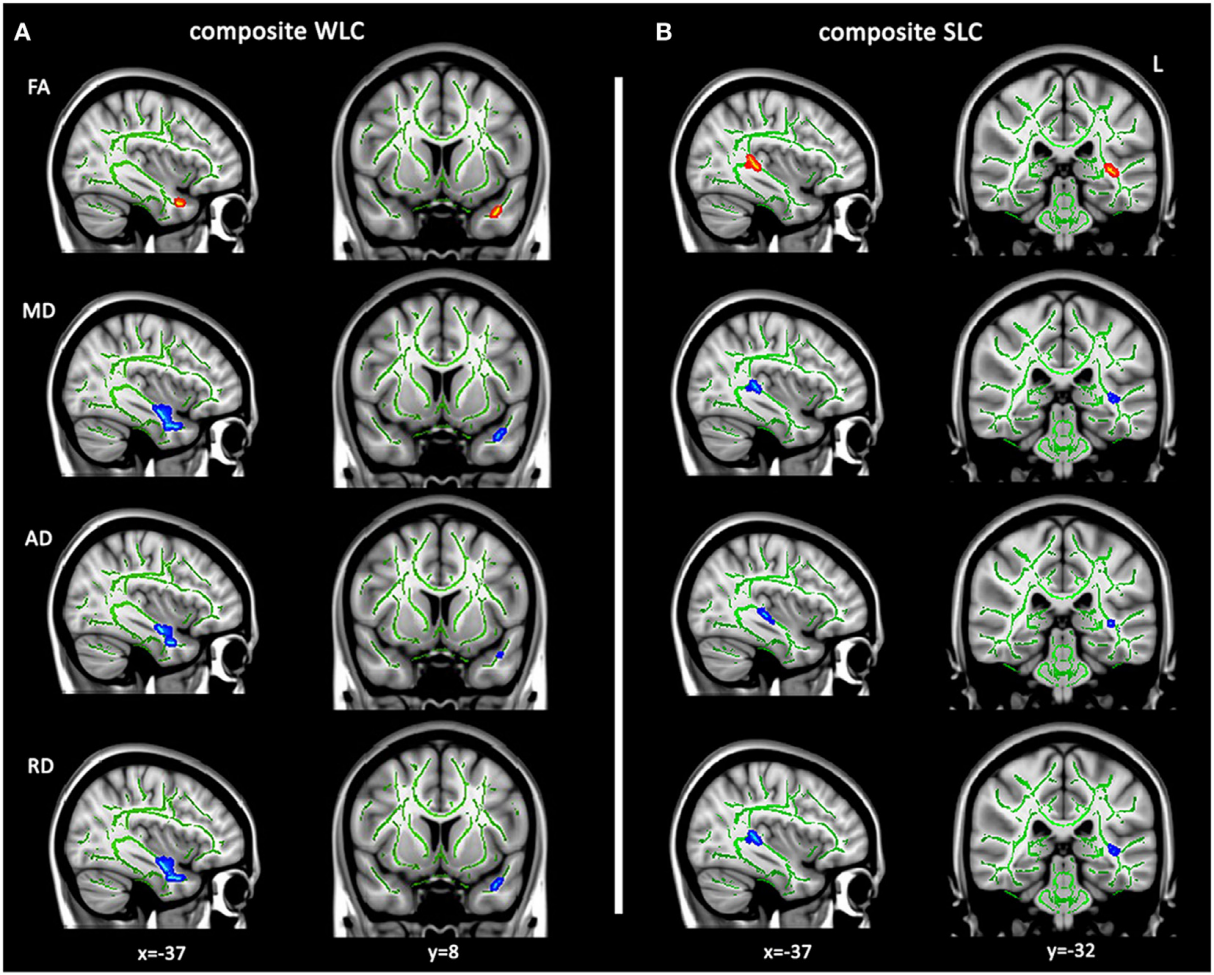
**Tract-based spatial statistics analysis for white matter integrity related to language comprehension**. Altered diffusion tensor imaging metrics related to composite WLC **(A)** and SLC **(B)** scores controlling for age and lesion volumes (TFCE corrected *P* < 0.05). Regions with significantly positive correlates of mean fractional anisotropy (FA) (red–yellow) or negative correlates of mean diffusivity (MD), axial diffusivity (AD), and axial diffusivity (RD) (blue–light blue) with individual comprehension scores are overlaid on the FMRIB58 FA skeleton (green), displayed on a template in Montreal Neurological Institute space. WLC, word-level comprehension; SLC, sentence-level comprehension.

Tract-based spatial statistics was conducted to localize areas in which white matter integrity related to WLC and SLC scores, controlling for age and total lesion volume. Because poor performance on word-level auditory comprehension tasks may relate to semantic impairment, rather than auditory comprehension *per se*, we added scores on the PPT test, a measure of non-verbal semantics, as an additional covariate for WLC. Because the SLC score already controlled for WLC (see Materials and Methods), further controlling SLC for non-verbal semantics was not necessary. TBSS analysis showed a strong association between WLC scores and DTI metrics (decreased FA and increased MD, AD, and RD) in a left anterior temporal region, which corresponds to a part of the ventral pathways where the IFOF, ILF, and UF run together (Figure 2A). SLC scores were significantly associated with DTI metrics in a left posterior temporal region, with decreased FA and increased diffusivities primarily located in parts of IFOF and ILF (Figure 2B). Notably, these results reflect only specific locations in which white matter integrity was associated with WLC or SLC scores. Thus, we next examined relationships between comprehension scores and DTI metrics of entire tracts implicated here.

### Relationship between White Matter Tracts and Comprehension Deficits

**Figure 3:**
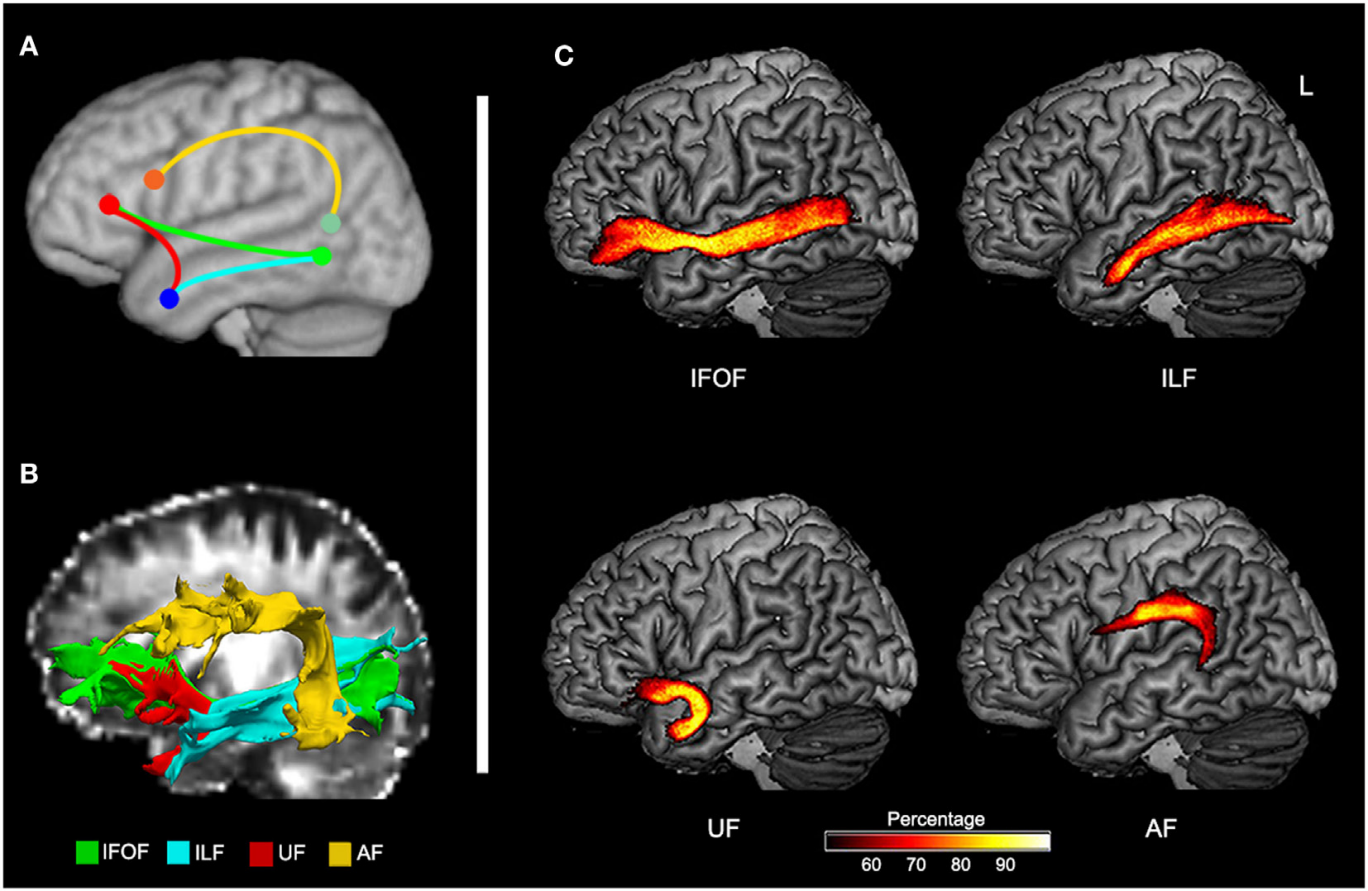
**Probabilistic maps of reconstructed ventral and dorsal pathways related to language**. **(A)** Schematic representation of expected tracts in Montreal Neurological Institute space. **(B)** Reconstructed inferior fronto-occipital fasciculus (IFOF), inferior longitudinal fasciculus (ILF), uncinate fasciculus (UF), and arcuate fasciculus (AF) in native space of a control subject. **(C)** Group-based probability maps of IFOF, ILF, UF, and AF derived from probabilistic tractography in healthy older subjects (at least 50% subjects of each voxel).

To define potentially relevant white matter pathways, the left ventral tracts implicated by the TBSS analysis, along with the AF (for completeness), were reconstructed in healthy older participants (Figure 3). First, we examined the relationship between direct lesion burden in each tract and comprehension. Partial correlations showed that when controlling for total lesion volume, the number of lesioned voxels in the AF and ILF related to SLC scores, while the number of lesioned voxels in the UF related to WLC scores (all corrected *P* < 0.05, Table 2).

**Table 2:** **Partial correlations between integrity of reconstructed tracts in left hemisphere and composite comprehension measures in patients controlling for age, total lesion volume, and lesions in each tract**.

Tract	Tract size (mm^3^)		Composite WLC					Composite SLC			
	
		Lesion	FA	MD	AD	RD	Lesion	FA	MD	AD	RD
IFOF	8,506	−0.214	0.410*	−0.447*	−0.489*	−0.432*	0.118	0.409*	−0.413*	−0.408*	−0.413*
ILF	5,451	−0.022	0.248	−0.378	−0.380	−0.369	−0.406*	0.382	−0.461*	−0.457*	−0.453*
UF	3,097	−0.400*	0.408*	−0.429*	−0.424*	−0.439*	−0.046	−0.010	−0.216	−0.232	−0.221
AF	3,447	−0.081	−0.127	0.129	0.110	0.138	−0.439*	0.074	−0.079	−0.088	−0.072

*IFOF, inferior fronto-occipital fasciculus; ILF, inferior longitudinal fasciculus; UF, uncinate fasciculus; AF, arcuate fasciculus; FA, fractional anisotropy; MD, mean diffusivity; AD, axial diffusivity; RD, radial diffusivity; WLC, word-level comprehension; SLC, sentence-level comprehension*.

*For lesion-performance analyses, total lesion volume was considered as covariate*.

*P < 0.05, Bonforroni corrections for multiple comparisons.

**Figure 4:**
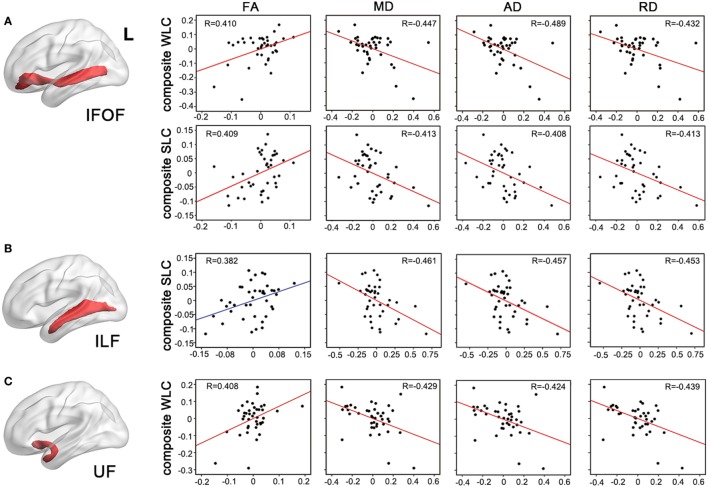
**Partial correlations between integrity of entire tracts and auditory comprehension**. Mean measures of white matter integrity [fractional anisotropy (FA), mean diffusivity (MD), axial diffusivity (AD), and axial diffusivity (RD)] of left inferior fronto-occipital fasciculus [IFOF, **(A)**], inferior longitudinal fasciculus [(ILF), **(B)**], and uncinate fasciculus [(UF), **(C)**] were associated with WLC or SLC composite scores, controlling for age, lesion volume, and lesioned voxels in the respective tracts. *P* < 0.05 with Bonferroni correction. WLC, word-level comprehension; SLC, sentence-level comprehension.

To test more specific measures of tract integrity, we next examined relationships between the comprehension scores and the average FA, MD, AD, and RD values in each tract. Partial correlations showed that when controlling for age, total lesion volume, and the number of lesioned voxels in each tract, reduced FA and increased diffusivity measures including MD, AD, and RD of the left IFOF were significantly associated with both WLC and SLC scores (corrected *P* < 0.05, Figure 4A). DTI metrics in the left ILF significantly related to the SLC scores, with significant correlations with MD, AD, RD, and a marginal correlation with FA (corrected *P* < 0.05, Figure 4B). In the left UF, DTI metrics significantly predicted the WLC scores (corrected *P* < 0.05, Figure 4C). When further controlling for PPT scores, correlations between the UF and WLC scores remained significant for MD, AD, and RD (all corrected *P* < 0.05), with a trend for FA (corrected *P* = 0.08). DTI metrics of the left AF did not relate to either WLC or SLC scores (all uncorrected *P* > 0.10). The full statistical results are shown in Table 2. Considering the potential confounding effect of handedness, we further performed the partial correlation analyses with the Edinburgh Handedness Index as an additional covariate and the results remained significant (data not shown). Taken together with the TBSS results, our findings showed that the anterior white matter connections *via* UF and IFOF were associated with WLC, while posterior white matter tracts including ILF and IFOF related to SLC.

## Discussion

We found that comprehension outcomes related primarily to damage in ventral stream white matter tracts in chronic left hemisphere stroke. Anterior temporal white matter integrity loss and involvement of the UF related primarily to word-level comprehension deficits. In contrast, posterior temporal white matter integrity loss and involvement of the ILF related primarily to sentence-level comprehension. Loss of white matter integrity in the IFOF related to both word- and sentence-level comprehension. Although lesion burden in the AF related to sentence-level comprehension, more specific examination of white matter integrity metrics revealed no relationship. Taken together, these findings are among the first to map critical white matter pathways for different aspects of auditory comprehension in chronic stroke.

The finding that word-level comprehension relies on anterior temporal white matter corresponds with recent fMRI findings in healthy participants (3) and gray matter atrophy findings in people with primary progressive aphasia (4), collectively demonstrating that the classical posterior localization of Wernicke’s area is incorrect. In line with these results, a previous study combining DTI and fMRI in a group of 10 patients with chronic post-stroke aphasia identified a relationship between UF damage and word-level comprehension (11). This study suggested that the left UF mediates semantic control during auditory word comprehension, by connecting inferior frontal cognitive control areas with anterior temporal areas storing word meanings. Another DTI study of post-stroke aphasia recently confirmed that lesion burden in left UF was associated with poor performance on word-to-picture matching and PPT tests, suggesting involvement of the left UF in semantic control during auditory word comprehension (10). We confirmed this prior result and extended it by showing that more specific measures of white matter integrity of the UF relate to word-level comprehension. Further, this relationship persists after controlling for non-verbal semantic performance on the PPT test, suggesting that the role of the UF in word-level comprehension does not simply reflect the role of the anterior temporal lobe in semantic knowledge (30), but that it plays a specific role in access to semantic information from auditory or perhaps amodal lexical representations.

The IFOF, like the UF, connects inferior frontal cortex to the temporal lobe. Whereas the UF connects to the anterior temporal lobe, the IFOF courses through the extreme capsule to posterior temporal areas, notably the middle temporal gyrus (31). We found that reduced IFOF integrity was significantly associated with both word- and sentence-level comprehension deficits. Previous lesion–symptom mapping studies implicated the posterior middle temporal gyrus in performance on both word and sentence comprehension tasks (31, 32). Although the authors suggested that involvement of the posterior middle temporal cortex in word-level comprehension could explain its relationship to deficits in all comprehension tasks, we found that the ventral stream connections between this area and the inferior frontal cortex *via* the IFOF relate not only to word-level comprehension but also to sentence-level comprehension after regressing out word-level deficits. Indeed, some studies in healthy subjects have found that auditory sentence comprehension is mediated in part by the IFOF (6).

These frontal–posterior temporal connections may play a similar role in cognitive control to the UF connections discussed above. Structural and functional imaging studies suggest that inferior frontal regions contribute to access of semantic knowledge, and hence auditory comprehension (33–35). Furthermore, damage to prefrontal regions leads to more severe deficits of semantic control than damage to temporo-parietal regions alone (36). Thus, the IFOF, with its long course from the inferior frontal lobe through the temporal lobe, may be involved in linking control areas of the frontal lobe to multiple levels of semantic representations in the temporal lobe (37). It should be noted that another study in stroke patients also found that disruption of left ventral fronto-temporal connections *via* extreme capsule fiber systems related to sentence comprehension, although the specific findings suggested a role in syntax rather than semantics (12). Our sentence comprehension tasks do not allow us to discriminate between syntax and semantics, but the role of IFOF demonstrated here in word comprehension tasks involving only concrete nouns that require no syntactic processing suggests a role in semantics. Coupled with the relationship to sentence-level tasks after controlling for word-level comprehension, these findings suggest that the IFOF plays a role either in multi-level semantic processing or in domain general processes underlying comprehension in general, such as cognitive control.

In addition to the IFOF, our results also implicated the ILF in sentence comprehension. The TBSS analysis suggested that an area of posterior temporal white matter including segments of both the ILF and IFOF was especially critical. Auditory sentence comprehension involves a number of different cognitive subprocesses and so engages a widely distributed network of frontal, temporal, and parietal areas (38). The most critical portion of white matter for sentence comprehension in the TBSS analysis here underlies posterior temporal areas implicated in semantics, phonology, syntax, and working memory (2, 37). Previous studies on healthy participants have shown that temporo-parietal areas activated during sentence comprehension participate in a number of white matter pathways including the IFOF and the ILF (39). The ILF connects the posterior temporal lobe with anterior temporal cortex, which has been implicated in both semantic (30) and syntactic processing (40). Evidence from fMRI studies of healthy participants has indicated that auditory sentence comprehension simultaneously activates both posterior and anterior temporal regions (41), implying involvement of anterior/posterior connections *via* the ILF. However, the exact nature of the processing supported by the ILF remains unclear. Additional research will be needed to examine this question further.

It has been well established that the lateral AF supports sensory-articulatory integration involved in speech production (6, 42, 43). DTI studies have implicated the AF in syntactic processing during sentence comprehension in children (44) and people with primary progressive aphasia (8). Here, we found that direct lesion load on left AF related to sentence comprehension in post-stroke aphasia, consistent with recent studies in this population (9). However, when controlling for direct lesion overlap on the AF, we found no association between DTI measures within AF and performance. This finding contrasts with a previous study showing that integrity within left AF and ventral extreme capsule fiber systems related to syntactic comprehension in stroke patients (12). However, this prior study did not control for direct lesion burden on the tracts, so integrity measures in this study may reflect both direct lesion damage as well as secondary axonal degeneration in the tracts. Different from this previous study, we factored out direct lesions to the tracts when examining white matter integrity metrics because the direct damage in a given tract likely covaries closely with damage in anatomically neighboring tracts. This covariance could result in false-positive findings in the tracts of interest. Factoring out the direct damage thus allowed us to examine the behavioral contributions of each white matter tract more specifically, by considering only the secondary degeneration. Two other considerations regarding the AF are worth noting. First, the prior studies above specifically examined syntax using tasks designed to control for semantics and other processes required for sentence comprehension. Since we focused on clinical tests, and not on disambiguating the subprocesses of sentence comprehension, our results may be less sensitive to a specific role of the AF in processing complex syntax. Additionally, the AF anatomically encompasses direct (fronto-temporal segment) and indirect (fronto-parietal or temporal–parietal segments) fiber bundles (42), and these bundles within AF may serve different functions in language processing (45). The roles of these different segments in auditory comprehension require further elucidation.

Several limitations should be noted. First, lack of objective measures of the individual tracts in patients is a main caveat of this study. Due to varied left hemisphere lesions, most of the left hemisphere white matter connections could not be reconstructed with probabilistic tractography in individual patients. Under this circumstance, white matter damage does not necessarily indicate that the adjacent cortical regions are crucial for comprehension behavior, but may rather indicate that connections between the posterior and the anterior cortical regions are associated with comprehension performance. Second, we did not determine the involvement of left hemisphere cortical areas in word or sentence comprehension. Previous studies have shown that regional gray matter damage in the left temporal lobe might support auditory comprehension processing in patients with post-stroke aphasia or primary progressive aphasia (4, 31, 32). Therefore, interrelationships between both cortical structures and white matter pathways involved in different levels of auditory comprehension need further investigation.

## Conclusion

The present findings implicate anterior temporal white matter and particularly the UF in word-level comprehension. In contrast, posterior temporal white matter damage and loss of integrity in the ILF related to sentence-level comprehension deficits. The IFOF, with its long course from the frontal lobe through the temporal lobe, was implicated in both word and sentence comprehension. These results demonstrate the importance of ventral stream white matter damage to auditory comprehension in stroke and suggest that anterior and posterior temporal white matter damage impairs different levels of auditory comprehension.

## Author Contributions

SX conceived the study, analyzed the data, and drafted the manuscript; EL contributed to the collection and analysis of the data; LS-K contributed to the data interpretation; PT contributed to the design of the study, the analysis and interpretation of the data, and editing of the manuscript. All the authors contributed to editing of the manuscript.

## Conflict of Interest Statement

The authors declare that the research was conducted in the absence of any commercial or financial relationships that could be construed as a potential conflict of interest.

## Footnotes

^1^http://www.fil.ion.ucl.ac.uk/spm.

^2^http://www.fmrib.ox.ac.uk/fsl/.
